# Analytical Workflows to Unlock Predictive Power in Biotherapeutic Developability

**DOI:** 10.1007/s11095-022-03448-y

**Published:** 2022-12-05

**Authors:** Markos Trikeriotis, Sergey Akbulatov, Umberto Esposito, Athanasios Anastasiou, Oksana I. Leszczyszyn

**Affiliations:** Research and Development, Malvern Panalytical Limited, Grovewood Road, Malvern, WR14 1XZ Worcestershire UK

**Keywords:** aggregation, colloidal stability, conformational stability, machine learning, monoclonal antibody

## Abstract

**Purpose:**

Forming accurate data models that assist the design of developability assays is one area that requires a deep and practical understanding of the problem domain. We aim to incorporate expert knowledge into the model building process by creating new metrics from instrument data and by guiding the choice of input parameters and Machine Learning (ML) techniques.

**Methods:**

We generated datasets from the biophysical characterisation of 5 monoclonal antibodies (mAbs). We explored combinations of techniques and parameters to uncover the ones that better describe specific molecular liabilities, such as conformational and colloidal instability. We also employed ML algorithms to predict metrics from the dataset.

**Results:**

We found that the combination of Differential Scanning Calorimetry (DSC) and Light Scattering thermal ramps enabled us to identify domain-specific aggregation in mAbs that would be otherwise overlooked by common developability workflows. We also found that the response to different salt concentrations provided information about colloidal stability in agreement with charge distribution models. Finally, we predicted DSC transition temperatures from the dataset, and used the order of importance of different metrics to increase the explainability of the model.

**Conclusions:**

The new analytical workflows enabled a better description of molecular behaviour and uncovered links between structural properties and molecular liabilities. In the future this new understanding will be coupled with ML algorithms to unlock their predictive power during developability assessment.

**Supplementary Information:**

The online version contains supplementary material available at 10.1007/s11095-022-03448-y.

## Introduction

Whilst improvements in technologies that aid discovery and optimisation of therapeutic proteins have increased the probability of finding effective molecules with a targeted biological activity, assessing whether these molecules have the capacity to successfully progress to clinical trials remains a complex and uncertain process [1–3]. This issue is compounded by the increasing numbers of next generation biotherapeutics entering drug development pipelines that do not fit existing platform approaches and come with a more limited understanding of their developability attributes [4]. Strategies for lowering the risk of advancing of candidate molecules and avoiding costly late-stage failures typically involve a suite of assays aimed at uncovering performance in key developability areas e.g. expression, solubility and stability. As well as excluding molecules with the least desirable properties, such assays inform further development activities to understand how the attributes of the lead molecule can be optimised and result in a drug product [3]. The scientific effort required to fully characterise the biochemical and biophysical attributes of candidate molecules such that further development is sufficiently de-risked is significant [5] and this experimental burden is likely to be intensified by a growing trend for more dimensions of developability to be evaluated earlier in the development process [6].

Several research papers have described developability processes implemented in specific industry laboratories [2, 7, 8], but a single set of assays that guarantees the successful development of a molecule does not yet exist. What these assays have in common is that each indicates on the performance of one dimension of developability that is RAG-rated (Red/Amber/Green) against historically defined thresholds specific to each laboratory. When undertaken for all dimensions of developability, this ‘traffic light’ system builds up into a collective understanding of developability performance across different molecules to enable the selection of the best performing molecules to the subsequent development stage. Engagement with the biopharmaceutical industry over the last five years has led to the observation that drug development scientists struggle with a ‘sea of ambers’, a situation where the measured assay parameters do not sufficiently differentiate the behaviour between molecules. In this situation the selection process increasingly relies on the expertise of the scientist to make judgements about the significance or meaning of one datapoint over another. In a seminal study of 137 clinical-stage antibodies, Jain *et al*. [9] observed redundancy in the data output from 12 developability assays which suggests that a degree of overlap exists in the information being gathered by the reported parameters across different assays. The presence of these types of relationships point to a significant limitation for the one-to-one mapping of an assay parameter with a specific developability risk and may explain the challenges experienced by the scientist during decision-making.

Despite the introduction of developability assessments as a strategy to de-risk development almost a decade ago [6], delivering new drug approvals remains an expensive and ultimately unsustainable activity [10–12]. This suggests that transformational changes rather than refinements to existing processes are needed to address these challenges. Exactly what form these transformational changes need to take is unknown, but a widespread interest in artificial intelligence (AI) in drug discovery [13] and the early promise of machine learning applied to developability-type problems [14–16] indicate that such methods could play a significant role here. The development of ‘eXplainable AI’ (XAI) [17–20] is seen as important to the acceptance of AI by practitioners in other scientific fields such as healthcare [21] and is likely to be a vital component of any AI-assisted method in the biopharmaceutical industry that has traditionally been slow to adopt innovations to processes due to safety and quality concerns [6]. Transparency, justification, informativeness and uncertainty estimation have been proposed as core elements of XAI in drug design [22] and this philosophy can be equally applied in the development of “white-box” models in the candidate selection process. However, harnessing the intuition, experience and expert knowledge [23, 24] of drug developers will be the principal factor in enabling useful and usable XAI-assisted models for developability.

Successful ML model development requires the availability of a high-quality training dataset formed of parameters that are relevant to the problem posed and enough datapoints to cover the variation that is likely to exist in the measured data. For a model that reliably predicts results from laboratory measurements, the challenge is to compile a training dataset with strong causation between the assay parameters and the property of interest whilst minimising the contribution of data with weak or no correlations [25]. The design of developability assays is one area where a deep and practical understanding of the problem domain would be advantageous for model accuracy and explainability, as the chosen parameters that form the model’s input are typically centred on a theoretical model of sample behaviour. Currently, there is a lack of published research on the interplay between assay and model designs, and the role that expert knowledge plays in understanding the relationships between input parameters for developability problems. We propose that expert knowledge can be incorporated into the model building process to improve relevancy and explainability in two ways: a) by combining or creating new input parameters from instrument or in silico data to better describe sample behaviour and b) guiding the choice of input parameters or machine learning techniques. In the first two case studies presented in this paper, we describe analytical workflows that use expert knowledge to bring new insight to commonly assessed developability risks and discuss how their design can conceptually influence machine learning aspirations. In a third case study we demonstrate how specific domain expertise can uncover relationships within the dataset that may lead to more accurate predictions.

## Materials and Methods

### Materials

The mAbs used in this study (Online Resource 1, Table S1) were produced by Evitria AG (Zurich, Switzerland) using a CHO (Chinese Hamster Ovary) expression system and were purified using Protein A chromatography. They were shipped under refrigerated conditions and once received they were aliquoted in 2 mL sterile cryogenic vials and stored at -80°C until use. All buffers were prepared using ultrapure water and analytical grade chemicals obtained from Fisher Scientific (Loughborough, UK).

### Sample Preparation

Each mAb was prepared in 15 different formulations arising from the combinations of five pH values (4, 5, 6, 7 and 8) with three NaCl concentrations (0, 50 or 150 mM). Buffer exchange was performed by dialysis using Xpress Mini Dialyzer MD1000 devices, MWCO 3.5 kDa (Scienova, Jena, Germany). Typically, ~ 400 µL of sample was dialysed at room temperature against 2.5 mL of target buffer, which was replaced every 30 min for a total of 6 cycles. After dialysis each sample was diluted to the target concentration for each characterisation technique and stored at 4–6°C until measurement. All measurements were completed within 24 h of the end of dialysis.

### Differential Scanning Calorimetry (DSC)

Measurements were performed on a MicroCal PEAQ-DSC Automated system (Malvern Panalytical Ltd., Worcestershire, UK). Each sample was diluted to 2.0 mg/mL and then measured relative to the corresponding buffer in the reference cell by applying a thermal ramp from 20°C to 110°C at a rate of 3.9°C/min. The thermal unfolding profiles were analysed with a custom script which calculated the onset of unfolding (T_onset_) and the transition midpoint temperatures (T_m_). The T_m_ values are either ordered by temperature (T_m_1, T_m_2, …) or assigned to structural features (T_m_(Fab), T_m_(C_H_2), T_m_(C_H_3)). The Fab region was assigned to the peak with the largest area and, from the remaining peaks, the C_H_2 domain was assigned to the peak with the lowest transition temperature [26].

### Dynamic (DLS) and Static (SLS) Light Scattering

The size, diffusion interaction parameter (k_D_) and thermal aggregation profile of each sample were measured using Light Scattering (LS) on a customised Zetasizer Ultra (Malvern Panalytical Ltd., Worcestershire, UK) adapted for automatic sampling from 96-well plates and for continuous monitoring of the scattering intensity during SLS thermal ramps. Size measurements using DLS were performed in quartz cuvettes at 25°C. Each sample was diluted to 2.0 mg/mL and run in triplicate with the attenuation and measurement process automatically optimised, while the scattered light was collected at 174.7^o^ angle. The Z-average diameter and polydispersity index (PDI) for each measurement were automatically calculated by the ZS Xplorer software.

To measure the diffusion interaction parameter (k_D_) a 5-point dilution series of each sample was prepared ranging from 1.0 to 5.0 mg/mL. The actual concentration of each diluted sample was calculated from its absorbance at 280 nm. Size measurements using DLS were performed for each diluted sample as described above. A custom script was used to combine the concentration and size measurements and output the k_D_, D_0_ (diffusion coefficient extrapolated to zero concentration) and the standard error of the linear fit (k_D__SE).

The thermal aggregation profile of each sample was acquired immediately after the size measurement was completed as described above. Samples were heated from 25°C to 90°C at a ramp rate of 3.9°C/min while the scattered light was detected at 90° angle. Custom scripts were used to extract two significant events from the thermal aggregation profiles: a) the earliest detectable sign of aggregation (T_agg_1) and b) the point where fast aggregation occurs (T_agg_2). The latter was defined as the temperature at which the scattering intensity passed an arbitrary threshold of 3,000 kcps which usually coincided with the fast aggregation phase of the thermal aggregation profile. This threshold was determined empirically based on the analyses of a large number of thermal aggregation profiles for various mAbs and it is specific to the instrument setup used in this study.

### Size Exclusion Chromatography (SEC)

SEC measurements were performed on a Waters H-class UPLC system (Waters Ltd., Wilmslow, UK) equipped with OMNISEC REVEAL UV–vis and RALS detectors (Malvern Panalytical Ltd., Worcestershire, UK). Samples were analysed using a Waters SEC column (Waters Xbridge Protein BEH, 3.5 µm, 7.8 × 150 mm) under a flow rate of 0.4 mL/min of mobile phase consisting of 50 mM sodium phosphate, 200 mM NaCl (pH = 6.8) at 25°C. Each sample was diluted to 2.0 mg/mL and run in triplicate injections of 3 µL. Sample compositions were obtained by analysing the chromatograms using a custom script which outputs the percent area of the Monomer and the Low and High Molecular Weight species (LMW, HMW) based on the UV signal at 280 nm.

### Structural Modelling and Electrostatic Potential Distributions of mAbs

3D structures for mAbs 1–5 were constructed by combining available Protein Data Bank (PDB) files of antibody fragments (Online Resource 1, Table S1). YASARA (YASARA, Vienna, Austria) and PyMol (Schrodinger, New York, USA) were used to process the PDB files and make corrections to match the actual amino acid sequence expressed. The full mAb templates of the IgG1 isotype were generated from available structures on PDB (1IGT, 1HZH and 5VGP) while for the IgG4 isotype the PDB files used were 5DK3 and 4C54. In the case of mAb-2, were only the Fv fragment was available, the remainder of the Fab was constructed by combining it with the mAb-5 file 2OSL, before generating the full mAb. After all corrections were performed, a final energy minimisation using the NOVA force field was applied. The electrostatic potential distributions were generated on YASARA using the Adaptive Poisson-Boltzmann Solver (APBS). The solver parameters were set to match the experimental conditions of the actual mAb characterisation by varying the pH from 4 to 8 and the ion concentration from 1 to 150 mM.

### Machine Learning (ML)

The initial dataset comprised 75 samples and values for 14 different parameters from DSC, LS, SEC and formulation. Of these, T_onset_, T_m_1, T_m_2, T_m_3 were set as target variables, with the remaining ten metrics used as predictors. To prepare the dataset for machine learning models, 15 samples with low quality measurements and 12 with missing values were removed. After excluding collinear features, the final, ML-ready dataset comprised 48 samples and 9 features (Online Resource 2). A direct multioutput regression approach was used to create independent regression problems for each target variable. To identify the best model for each temperature, many different algorithms were trained and cross-validated using the AutoML framework from H2O.ai python module [27]. These included Generalised Linear Models (GLM), Random Forest (RFs), Gradient Boosting Machines (GBMs) including but not limited to XGBoost, and Neural Networks with Multi-Layer Perceptron architecture (MLP). The best classes of models (GBM and MLP) were advanced to a stage of intensive hyperparameters training using the python libraries Optuna [28] and sklearn [29]. For every model in the above pipeline, five-fold cross-validation was used and paired with five random repetitions, which generated a total of 25 train-validation splits. The model’s performance was then assessed by averaging the Mean Absolute Error (MAE) between measured and predicted values over the validation sets.

## Results and Discussion

### Case Study 1 – Site-Specific Aggregation Propensity

Thermal unfolding and thermal aggregation profiles are information-rich data sources that are frequently used to screen for stability risks and those risks associated with stress conditions during developability assessments [2]. In current practice, the temperature of thermal unfolding (T_m_) calculated from thermal unfolding profiles is used to inform on the conformational stability. A higher T_m_ value is desirable as it has been correlated to better long-term stability and lower aggregation rates during accelerated stability studies [2, 30, 31]. Unfolding of the Fab region was found to be a better predictor of stability than unfolding of other domains [31] and is frequently used for ranking and selecting candidates for further development. Thermal aggregation profiles are indicative of the colloidal stability of the partially or fully unfolded molecules. During these measurements, there is usually a timepoint at which a substantial increase in scattering intensity is detected due to aggregation of a non-native form of the mAb molecule and the corresponding temperature is known as T_agg_, or onset aggregation temperature.

Although both types of thermal stability measurements are often performed together to provide a complementary characterization of sample attributes [26], the assay data are usually treated as independent monitors of different risks. Whilst this reductionist approach has enabled a process where candidates can advance through the development pipeline, failures due to unforeseen and undesirable molecular behaviour still occur downstream. These failures can be viewed as risk factors that are present in the developability landscape for antibodies but are not adequately monitored by measured parameters in existing assays. Predicting these parameters may lower the experimental effort and decrease the cycle time but the probability of technical success would not be improved unless the data collected better describes as many of the risk factors that are likely to be encountered downstream. In this study, we outline an analytical workflow that combines thermal stability data to identify additional molecular liabilities that would otherwise remain invisible by current analysis methods used in developability.

**Fig. 1:**
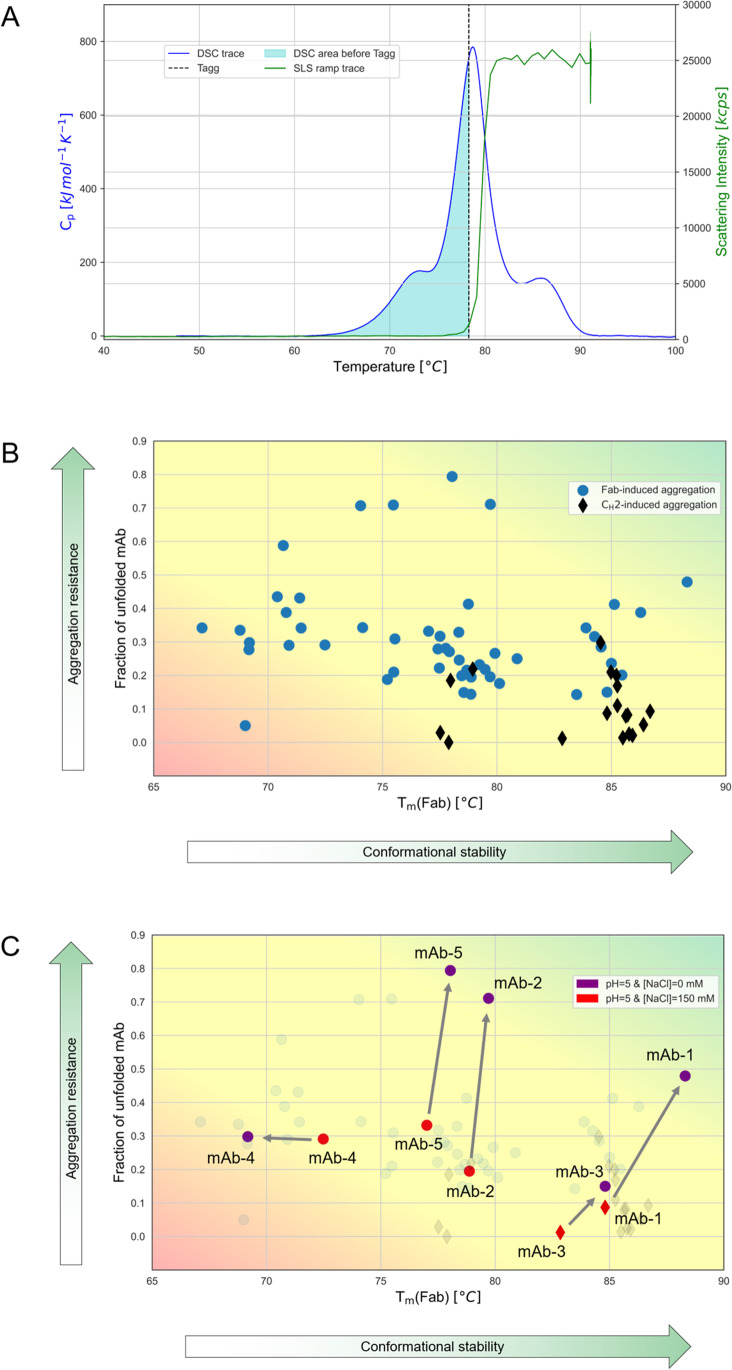
(**A**) A representative overlay of thermal unfolding (blue) and aggregation (green) profiles for mAb-5 (pH = 6, [NaCl] = 0 mM). The dotted line indicates the aggregation onset temperature, T_agg_. The blue shaded area corresponds to the fraction of unfolded protein in the sample before the aggregation onset temperature is detected. (**B**) A plot of developability attributes derived from the thermal profile overlays for the 75 samples characterised in this study. Blue circles indicate aggregation induced by unfolding of the Fab region, black diamonds indicate aggregation induced by unfolding of the C_H_2 domain. (C) A case study: developability attributes for mAb-1 – mAb-5 measured in the same conditions (red circles and diamonds: pH = 5, [NaCl] = 150 mM) showing potential liabilities for each molecule (conformational, aggregation propensity and site-specific aggregation); changing formulation (purple circles: [NaCl] reduced to 0 mM) can improve, or in case of mAb-4 worsen, the developability attributes of the molecules.

From the full dataset collected for the five mAbs, the new analytical workflow utilises the DSC and SLS thermal ramps measured under closely matched experimental conditions. The resulting raw measurement data was overlaid to simultaneously visualise the unfolding and subsequent self-assembly events that occur during heating (Fig. 1A). From this, we identify three important features that characterize sample behaviour: a) the temperature of thermal unfolding of the Fab region, T_m_(Fab), which we take to represent the overall conformational stability; b) the mole fraction of globally unfolded protein [32] at the point when aggregation is first detected (shaded area in Fig. 1A), which provides information about the reactivity of aggregation prone regions; and c) the domain assignment of the unfolding transition that coincides with the point when aggregation is first detected (Online resource 3).

The information coming from the thermal unfolding and thermal aggregation profiles was plotted together to draw out a holistic picture of the thermal stability landscape for the mAbs characterised in our study (Fig. 1B). The three features that describe the sample behaviour are included in this visualisation. First, the T_m_(Fab) forms the X-axis and indicates the conformational stability of each sample; the values vary between 68 – 88°C in our dataset. Second, the fraction of unfolded mAb at the time aggregation starts is plotted on the Y-axis. The interpretation of this metric is based on the fact that when an antibody solution is heated, conformational changes expose buried hydrophobic patches and initiate hydrophobic interactions between molecules. These hydrophobic interactions are the main driver behind thermally induced aggregation [14, 33, 34]. Across the studied samples the values on this axis vary between 10 – 80%. A situation where only a small fraction of unfolded protein corresponds with detectable aggregation is undesirable and indicates high aggregation propensity due to the presence of at least one highly reactive, aggregation prone region that can be easily exposed to the surface. Finally, the structural resolution afforded by the DSC thermograms enabled the locations of the aggregation prone regions to be identified. This is shown with either hollow or filled symbols indicating aggregation via the Fab region or C_H_2 domain, respectively (Fig. 1B). We observed that in roughly 30% of cases, aggregation was detected during the unfolding of the C_H_2 domain, and this agrees with reports suggesting that the aggregation of antibodies can be mediated by unfolding of either the Fab region or C_H_2 domain or both [34, 35].

To exemplify how this type of plot could be used for assessing liabilities and identifying optimization strategies in a panel of candidate molecules, we present a case study that includes all the samples that were measured at pH = 5 (Fig. 1C). A conventional approach would use T_m_(Fab) as the primary metric in order to rank these mAbs while in the new analytical workflow samples with desirable behaviour are identified by their location to the top right of this plot. Therefore, while the conventional approach would identify mAb-1, with T_m_(Fab) = 84°C, as the candidate displaying the most desirable biophysical behaviour, two molecular liabilities would be missed. These are uncovered in the new approach: a) the presence of highly reactive aggregation prone regions, which is inferred from the small fraction (< 0.1) of unfolded mAb required to detect aggregation in this sample; and b) the aggregation of the sample starting with the unfolding of the C_H_2 domain, which precedes the unfolding of Fab region. These liabilities do not necessarily mean that mAb-1 should be discarded from further development, rather a better formulation can be found to tackle both liabilities. For example, in the absence of NaCl the conformational stability is improved (T_m_ increases from 84 to 88°C), aggregation caused by the C_H_2 domain is suppressed, and the aggregation resistance is substantially increased (fraction unfolded increases from 0.09 to 0.48) moving it towards more desirable behaviour. The same type of analysis and development of mitigation strategies can be applied to the other candidates. Both aggregation resistance and conformational stability for mAb-2, mAb-3 and mAb-5 can be improved by removing NaCl, but the formulation of mAb-4 would also require a pH change to significantly improve aggregation resistance.

**Fig. 2:**
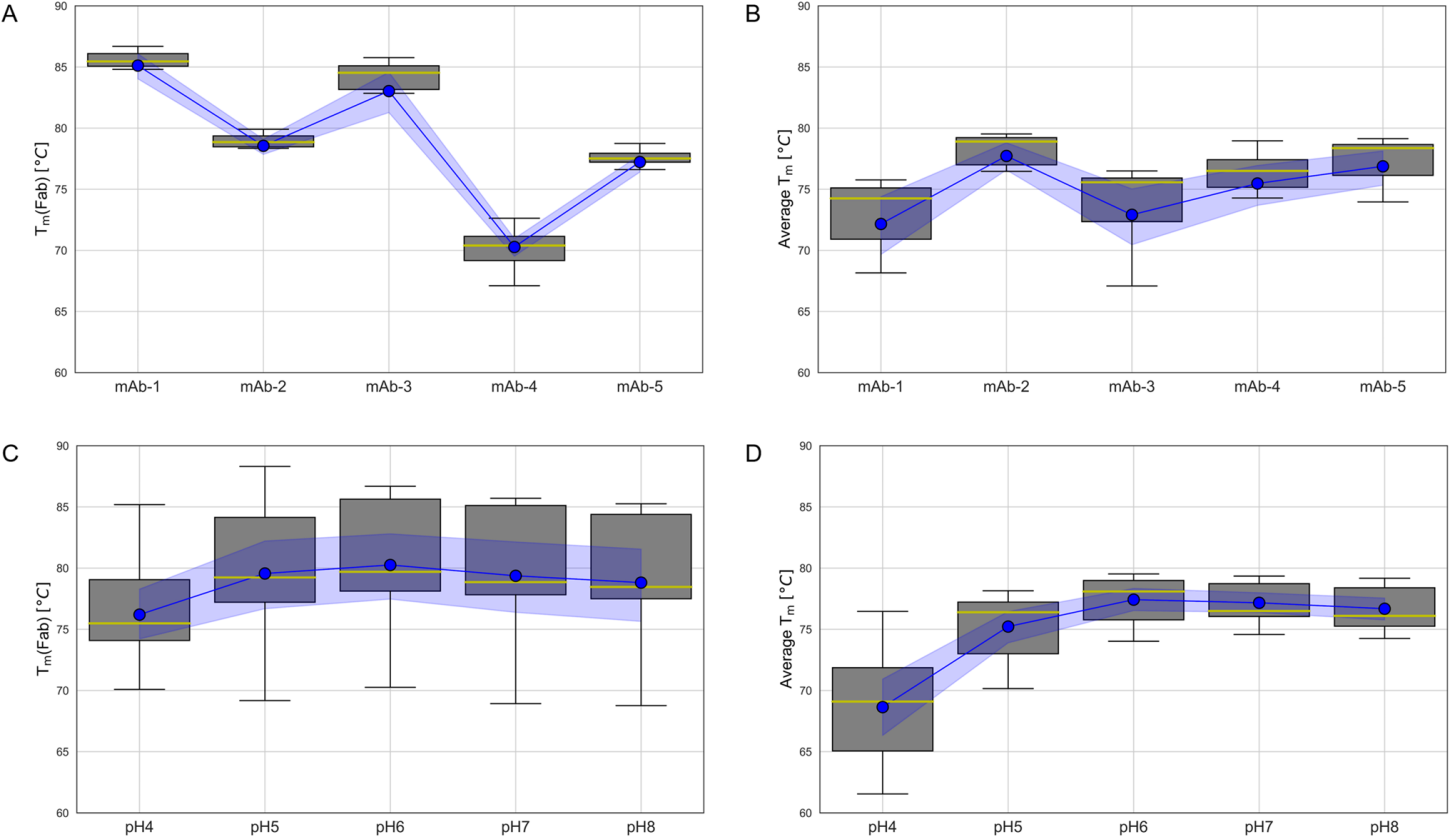
Box and whisker plots illustrating the trends and distribution of measured values in the thermal stability dataset: (**A**) T_m_(Fab) grouped by mAb studied; (**B**) average T_m_ by mAb studied; (**C**) T_m_(Fab) grouped by pH; (**D**) average T_m_ grouped by pH. In each box and whisker plot, the box represents the interquartile range (Q1-Q3), the horizontal green line inside it the median value, the blue dot the mean value and the shaded area the standard deviation of the mean.

In its present form, this analytical workflow could already be applied to mAb molecules outside of this study provided that the pre-requisite experimental data was available. However, some or all the data required for this analysis could be predicted from measured values of orthogonal techniques using a ML approach (see Case Study 3) or from amino acid sequences. Looking more closely at the distribution of the data reveals some factors that would need to be considered as part of a future ML strategy. The values of T_m_(Fab) are highly stratified with minimal overlap across the different molecules (Fig. 2A). For example, T_m_(Fab) values for mAb-4 make up 100% of the datapoints between 67 and 73°C while mAb-5 values make up 76% of datapoints between 74 and 78°C. This highly segregated data indicates a very strong relationship between the absolute T_m_(Fab) values and some specific aspect of these molecules that is not readily shared between them. In small datasets, the impact of such artefacts on predictive accuracy manifests as a lack of model generalisability; a situation that was also evidenced in the robustness of previously reported models [14]. Feature transformation – where the data is modified but the information is kept intact – is one way to counteract these effects. Using the average T_m_ values across molecules not only normalises the distribution of these values across the mAb molecules (Fig. 2C), but also reveals a relationship between pH and T_m_ (Fig. 2D) that was otherwise hidden (Fig. 2B). Making these trends more obvious not only to the algorithm but also to the expert will undoubtedly be a necessary step to satisfying both predictive accuracy and explainability requirements.

### Case Study 2 – Salt Effects Reveal Colloidal and Conformational Liabilities

In this case study we propose a novel analytical workflow that explores the effects of salt concentration on the colloidal and conformational stability of mAbs and uncovers liabilities that may lead to aggregation. Protein aggregation occurs through self-assembling that is often, but not always, preceded by partial or global unfolding of the protein structure. Aggregation may also be initiated by chemical degradation, nucleation on interfaces or direct self-assembly of the native protein [36, 37]. Salt concentration, along with other environmental parameters such as pH or excipients, affects these unfolding and self-assembly steps. This occurs either through Hofmeister effects [38–40] that modulate the interaction between water molecules and dissolved ions with the protein surface, or through salt screening effects that change the thickness of the electric double layer that surrounds the protein and attenuate electrostatic forces [41–45].

**Fig. 3:**
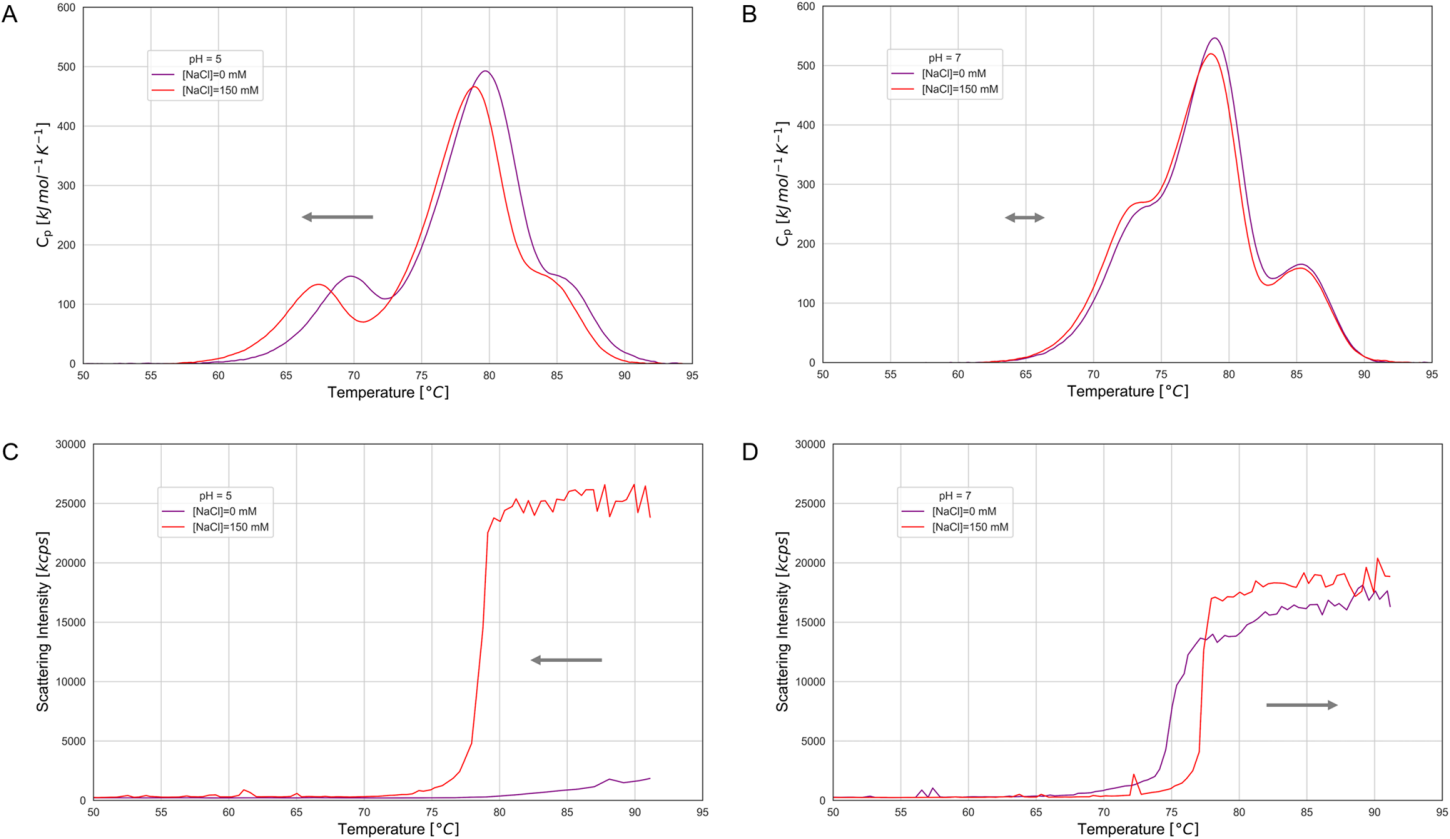
Observation of salt effects on thermal unfolding profiles (**A** and **B**) and thermal aggregation profiles (**C** and **D**) of mAb-2. Results from experiments at pH = 5 are shown in panels **A** and **C** while results from experiments at pH = 7 are shown in panels **B** and **D**. The arrows indicate the direction of the observed shift after the addition of 150 mM NaCl compared to no additional salt; the double arrow on panel B indicates that no significant shift was observed. (kcps: kilo counts per second).

In practice, the new workflow combines the data from DSC and SLS experiments in low and high salt conditions to observe the effects of salt to the unfolding and aggregation profiles. As an example, the thermal profiles of mAb-2 reveal that it unfolds at lower temperatures in the presence of 150 mM NaCl compared to no additional salt (Fig. 3). However, this effect is more pronounced at pH 5 compared to pH 7. The T_m_(C_H_2) at pH 5 shifts 2.4°C lower while at pH 7 the shift is less than 0.3°C. The addition of salt has a bigger impact on the observed aggregation temperature, T_agg_2. Interestingly, opposite salt effects are observed at pH 5 and 7. While at pH 5 T_agg_2 is shifted significantly lower when 150 mM NaCl are added, the shift is in the opposite direction at pH 7 with T_agg_2 being 2.6°C higher in the presence of 150 mM NaCl.

This last observation, where the addition of salt causes an increase in T_agg_2, can help us explain the behaviour of mAb-2 at pH7. There are two hypotheses that would explain the observed increase of T_agg_2: a) adding salt increases the structural stability so that unfolding happens at higher temperature and then aggregation follows; b) adding salt increases the colloidal stability so that self-assembly is delayed. The first hypothesis can be rejected since the unfolding profile of mAb-2 at pH 7 stays unaffected by the addition of salt. Therefore, an increase in colloidal stability is the only remaining explanation and this leads us to conclude that the observed increase of T_agg_2 is driven by electrostatic screening that neutralises attractive forces. Although this attraction is observed at high temperatures where at least partial unfolding has occurred, it is expected to be present at lower temperatures as well because of its electrostatic nature. Therefore this workflow, based on salt effects, can reveal inherent liabilities of mAbs caused by electrostatic attraction.

The observation of salt effects on the DSC thermograms can also provide information about the conformational stability. Across the 25 samples measured, the salt effects on the thermograms varied in both magnitude and direction of shift. Although the direction may be holding valuable information, we propose that the magnitude of the shift is more important. A sample that shows large shifts can be interpreted as having a flexible structure that can be easily affected by its environment causing it to adopt different conformations while the opposite can be interpreted as structural robustness. In the new analytical workflow, the observation of large shifts due to changing salt concentration reveals an inherent conformational liability for that sample.

**Fig. 4:**
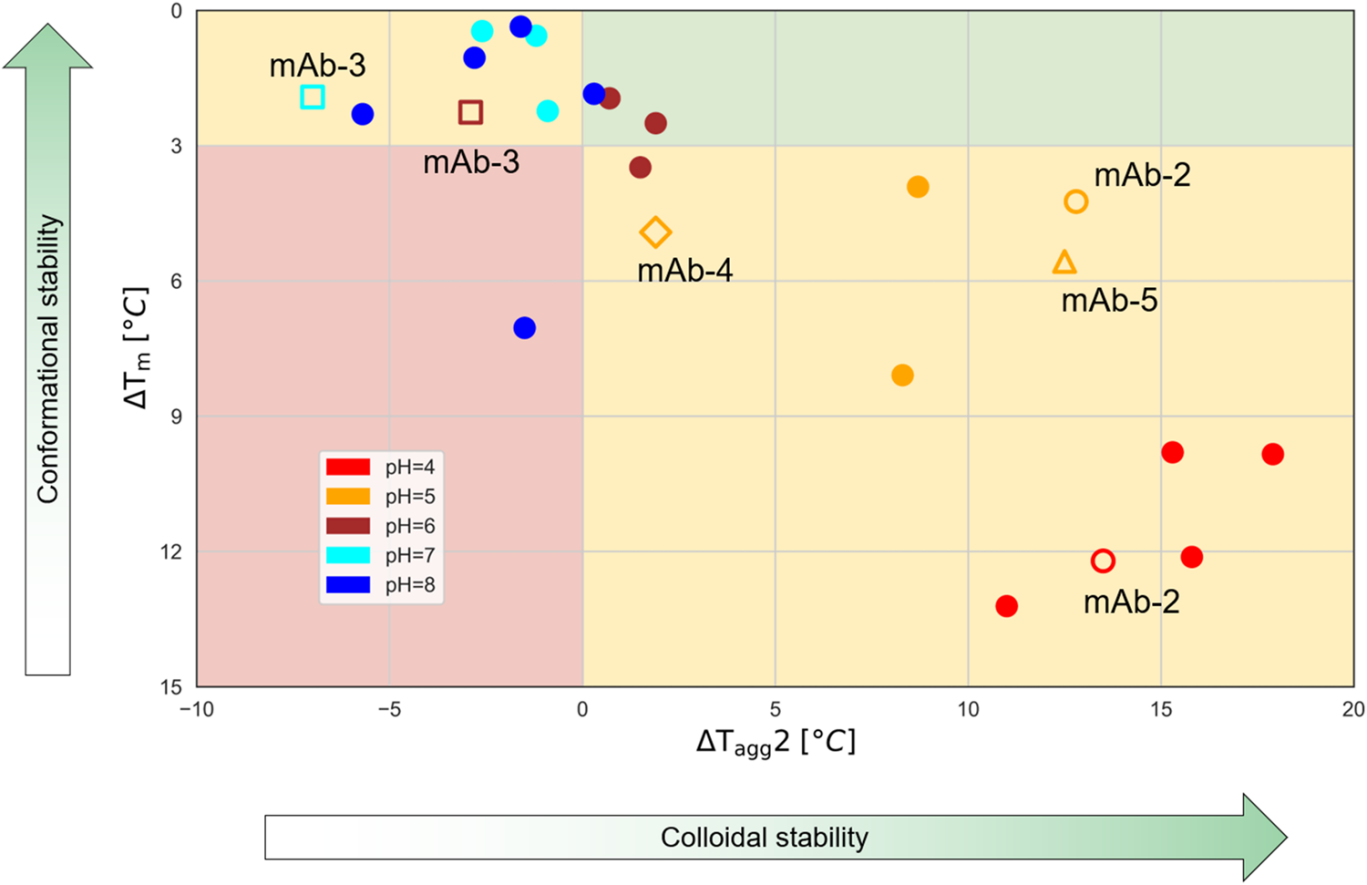
Salt effects plot for the 5 mAbs measured at 5 different pH values. The colouring of the data points represents the buffer pH as indicated on the legend. The four quadrants on the plot are shaded to indicate liabilities: red shading indicates samples with both colloidal and conformational liabilities; amber shading indicates samples with either colloidal or conformational liability; green shading indicates samples with no liabilities. Open shapes highlight samples that are discussed in the text: mAb-2 at pH 4 and 5 (open circles), mAb-3 at pH 6 and 7 (open squares), mAb-4 (open diamond) and mAb-5 (open triangle) at pH 5.

To capture the complex information arising from the combination of two analytical techniques and two different salt concentrations we have chosen a visualisation that shows both the colloidal and conformational stability of a sample in a single plot (Fig. 4). ΔT_agg_2 is the salt effect on T_agg_2 and is plotted on the X-axis, while ΔT_m_, the salt effect on T_m_, forms the Y-axis. The definition of ΔT_agg_2 and ΔT_m_ and the calculated values can be found in Online Resource 1 and Table S2. Negative values on the X-axis mean that the T_agg_2 increases with the addition of salt and therefore indicate a colloidal liability due to electrostatic attraction. In the case of the Y-axis higher values for the ΔT_m_ mean a larger shift in different salt concentrations and therefore a higher conformational liability due to increased structural flexibility. Moreover, we have set thresholds on the two axes of the plot to form four separate areas that indicate different sample behaviour. For the X-axis it is reasonable to split into negative and positive values; the negative values indicate a colloidal liability while positive values indicate colloidal stability. In the direction of the Y-axis an arbitrary threshold of ΔT_m_ = 3°C was chosen to split into structural flexibility (high ΔT_m_) and structural robustness (low ΔT_m_).

A significant observation from this analysis is that the majority of samples fall on the two quadrants where there is either a colloidal or a conformational liability, while the other two quadrants are sparsely populated. Several mechanisms driving the effect of different salts and excipients on protein stability have been proposed, including changes in the water structure, preferential binding or exclusion from the protein surface or competition for hydration between the protein and ions or excipients [40, 42, 46, 47]. What all the various theories have in common is that they observe a trade-off between conformational and colloidal stability. For example, kosmotropic anions in the Hofmeister series, such as SO_4_^2−^, promote conformational robustness while causing colloidal instability as observed in precipitation via salting-out. In a similar manner, excipients that preferentially bind on a protein’s surface favour colloidal stability but promote conformational destabilisation at the same time [45]. This trade-off between colloidal and conformational stability is clearly seen in the salt effect plot (Fig. 4). Interestingly, samples that were measured at low pH (pH = 4–5) populate the lower right quadrant that indicates high colloidal but low conformational stability. When moving close to neutral pH, and closer to the pI of each mAb, the balance shifts towards better conformational rather than colloidal stability and the samples now appear at the top left quadrant.

In addition to the value of the salt effect plot in understanding the liabilities of a sample, it can also be a valuable tool in devising a strategy to optimise its behaviour. To demonstrate this we present two examples where changing the buffer pH reduces the mAb’s liabilities. First is the example of mAb-2, which exhibits high colloidal stability at pH 4 due to the positive net charge it is expected to have at low pH (Fig. 4). However, its conformational stability is low as indicated by the high T_m_ shift (ΔT_m_ = 12.2°C). Changing the pH of this sample to 5 results in a significant increase in conformational stability with ΔT_m_ = 4.2°C, only slightly below the arbitrary border between robust and flexible structure. This change in pH, however, doesn’t impact the colloidal stability and the repulsive electrostatic forces remain present. The second example is mAb-3 at pH 7, the leftmost sample on the plot which means it has the lowest colloidal stability. The observation of -7.0°C ΔT_agg_2 on the X-axis indicates strong attractive forces. However, changing the pH to 6 reduces the ΔT_agg_2 by more than half without affecting its conformational stability as shown by the small change in ΔT_m_.

**Fig. 5:**
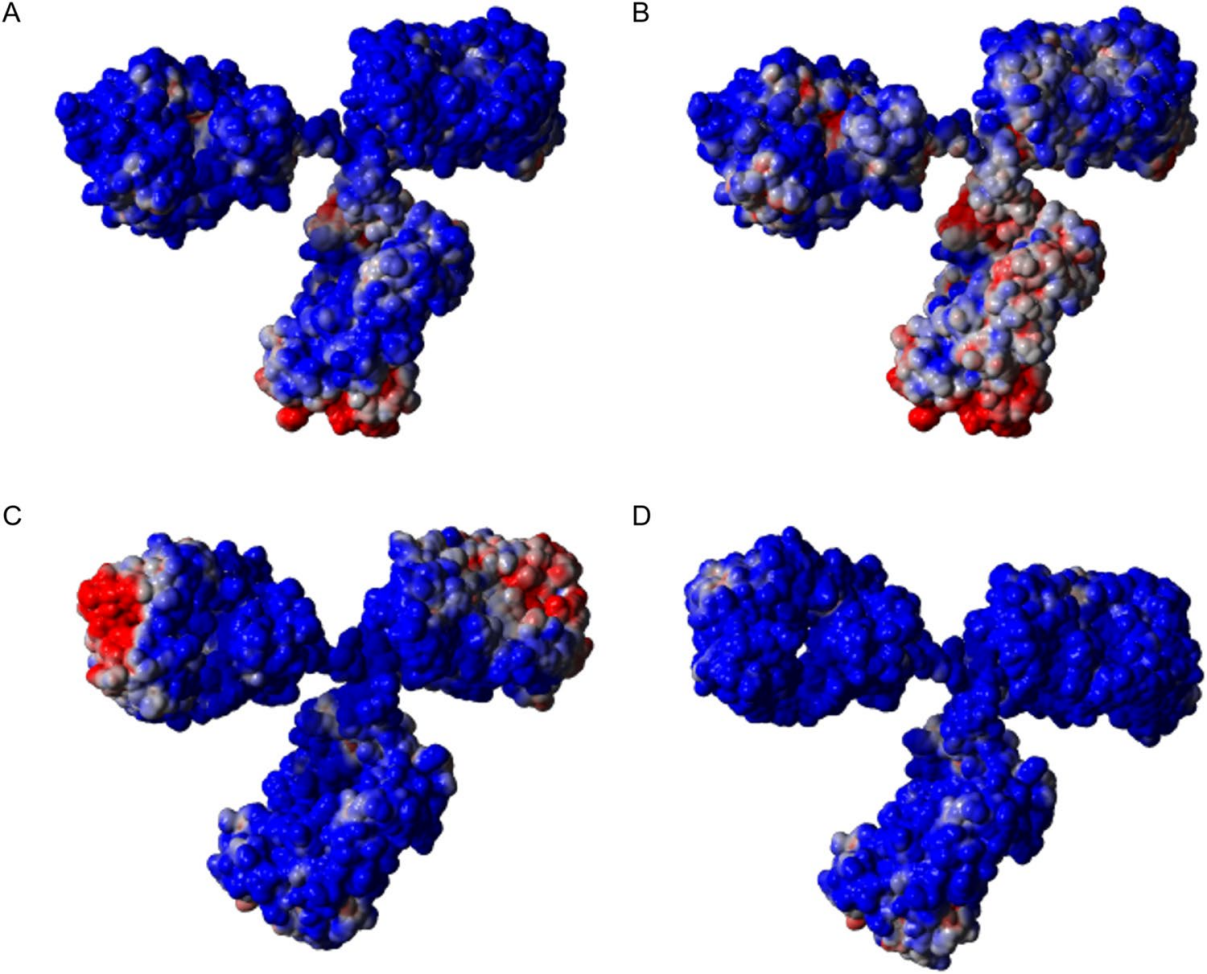
Charge distribution maps of selected mAbs. Areas with positive charge are shown in blue and areas with negative charge in red. (A) mAb-3 at pH 6; (B) mAb-3 at pH 7; (C) mAb-4 at pH 5; (D) mAb-5 at pH 5.

This last example also shows how the predicted liabilities from this analytical workflow can be linked to structural features of the mAb. The charge map of mAb-3 at pH 7 (Fig. 5B) reveals a polarised charge distribution with the Fc region of the mAb being negatively charged while the Fab region is positively charged. This is not the case at pH 6 where the distribution is evenly positive except for the C_H_3 domain (Fig. 5A). This is in agreement with the theoretical pI values for mAb-3: 6.04 and 7.95 for the Fc and Fab regions respectively (Online Resource 1, Table S1). This results in a negative net charge for Fc and a positive net charge for Fab at pH = 7. This polarised charge distribution, which has been previously reported for antibodies of the IgG-4 subclass [43], is a liability because there is the potential for dimerization due to attractive forces between the Fc and Fab regions of two individual mAb molecules. Therefore, the charge maps of mAb-3 verify the predicted liability from the new analytical workflow.

An additional example comes from the comparison of the charge distributions and behaviour of mAb-4 and mAb-5 at pH 5 (Fig. 5C and 5D). It is evident that the Fv region of mAb-4 is strongly negatively charged in contrast with the rest of the molecule, while mAb-5 has a uniformly distributed positive charge. The negatively charged Fv is a liability for mAb-4 because it increases the risk of dimerization due to electrostatic attraction. This is predicted on the salt effect plot by the fact that mAb-5 is further to the right compared to mAb-4 with a ΔT_agg_2 for the former at 12.5°C and for the later at 1.9°C.

These final two examples demonstrate how liabilities can potentially be predicted directly from the amino acid sequence and structural models. Although this predictive capability is not within reach yet, this new analytical workflow that utilises the salt effect observations to predict the solution behaviour of mAbs, is a first step in that direction. As our understanding of protein behaviour is used to enhance the capabilities of computational tools, we build strong foundations for the development of predictive solutions for mAb developability.

### Case study 3 – Prediction of T_m_ Using Machine Learning

Whilst a wider spectrum of assays is needed to better characterise mAbs and their liabilities, many of the assays used in developability show a certain degree of overlap [9]. This redundancy implies that the various assays probe aspects of molecular behaviour that are not completely independent, which opens the possibility of using ML to predict some of the developability parameters that are in use. However, the ability of a ML model to make good predictions is limited to the sort of parameters used to train the model. Ideally, only a reduced set of orthogonal features should be needed to fully describe the mAbs developability space, and a model trained on these features should be able to accurately predict any other redundant parameter. The challenge is to identify which assays provide such parameters, and here is where coupling ML techniques with the knowledge of expert users is of paramount importance. It is fundamental that knowledge transfer flows in both directions: not only expert users assisting with the design of experiments and models to improve the quality of the inputs provided, but also the ability to learn from the model by understanding how predictions are made [24, 48–50]. In recent years, there has been a growing awareness around the importance of building models that are either interpretable or explainable [19]. Here we present our first attempt to simplify the experimental characterisation of mAbs by showing that DSC-related parameters can be predicted from combined measurements of SEC and LS.

The synergy between modellers and domain experts was utilised to apply a quality filter to the data at the level of the instruments and techniques used. Based on this, SEC data for mAb-1 was classed as below standard and excluded for this study (Online Resource 2). Additional filters were applied on numerical and statistical level to further remove samples presenting missing values. A high confidence dataset was obtained through this procedure that not only was ready for ML models but also curated and enriched by expert knowledge. This dataset was used to train ML models aiming to predict the four major transition points observed in thermograms of mAbs: T_onset_, T_m_1, T_m_2, T_m_3. Having four target metrics meant constructing four different, independent regression models, each predicting one temperature using the same set of input metrics obtained from LS and SEC in addition to sample metadata (pH and salt concentration).

**Fig. 6:**
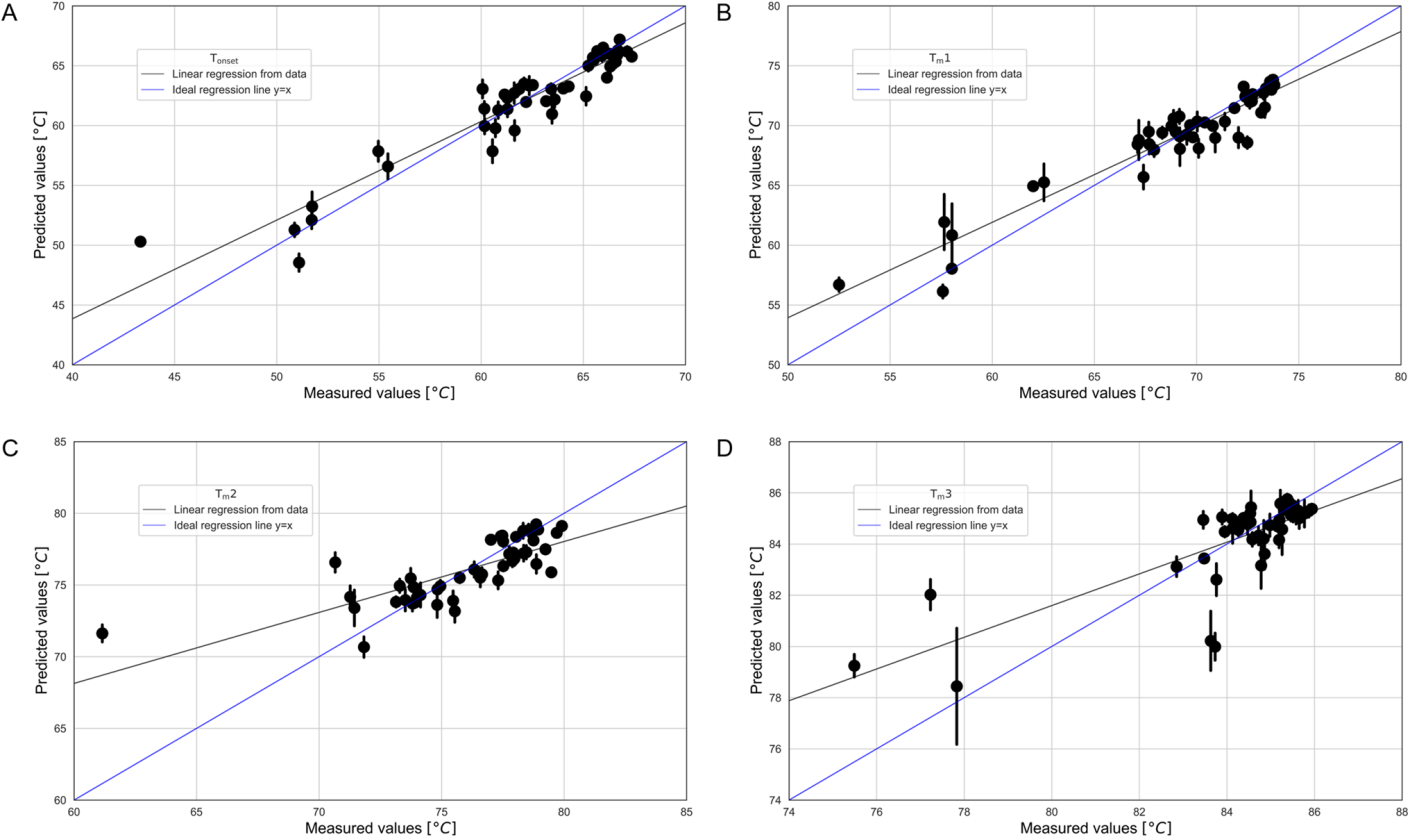
Plot of measured *vs* predicted values for each of the four temperatures predicted by the ML models. Black vertical bars represent the standard error across the cross-validation folds. Ideal behaviour is depicted by the y = x blue line, whereas the black line is the result of a linear regression between measured and predicted values. The closer the linear regression is to the ideal line, the more accurate the predictions are. (**A**) T_onset_ model: Pearson correlation: 0.95; Mean Absolute Error: 1.25; (**B**) T_m_1 model: Pearson correlation: 0.95; Mean Absolute Error: 1.16; (**C**) T_m_2 model: Pearson correlation: 0.74; Mean Absolute Error: 1.41; (**D**) T_m_3 model: Pearson correlation: 0.81; Mean Absolute Error: 0.77.

For each target temperature, several models were trained with fivefold cross-validation and their hyperparameters were optimised by minimising the average Mean Absolute Error (MAE) over the validation sets. Gradient Boosting Machines (GBMs) were ultimately chosen, not only because of their high performance but also because they allow for a more transparent model as opposed to black box techniques like neural networks. The predictions obtained with the best GBM for each of the target metrics is shown in Fig. 6. The high performance of each of the models was certified by high correlation values and low MAE.

**Fig. 7:**
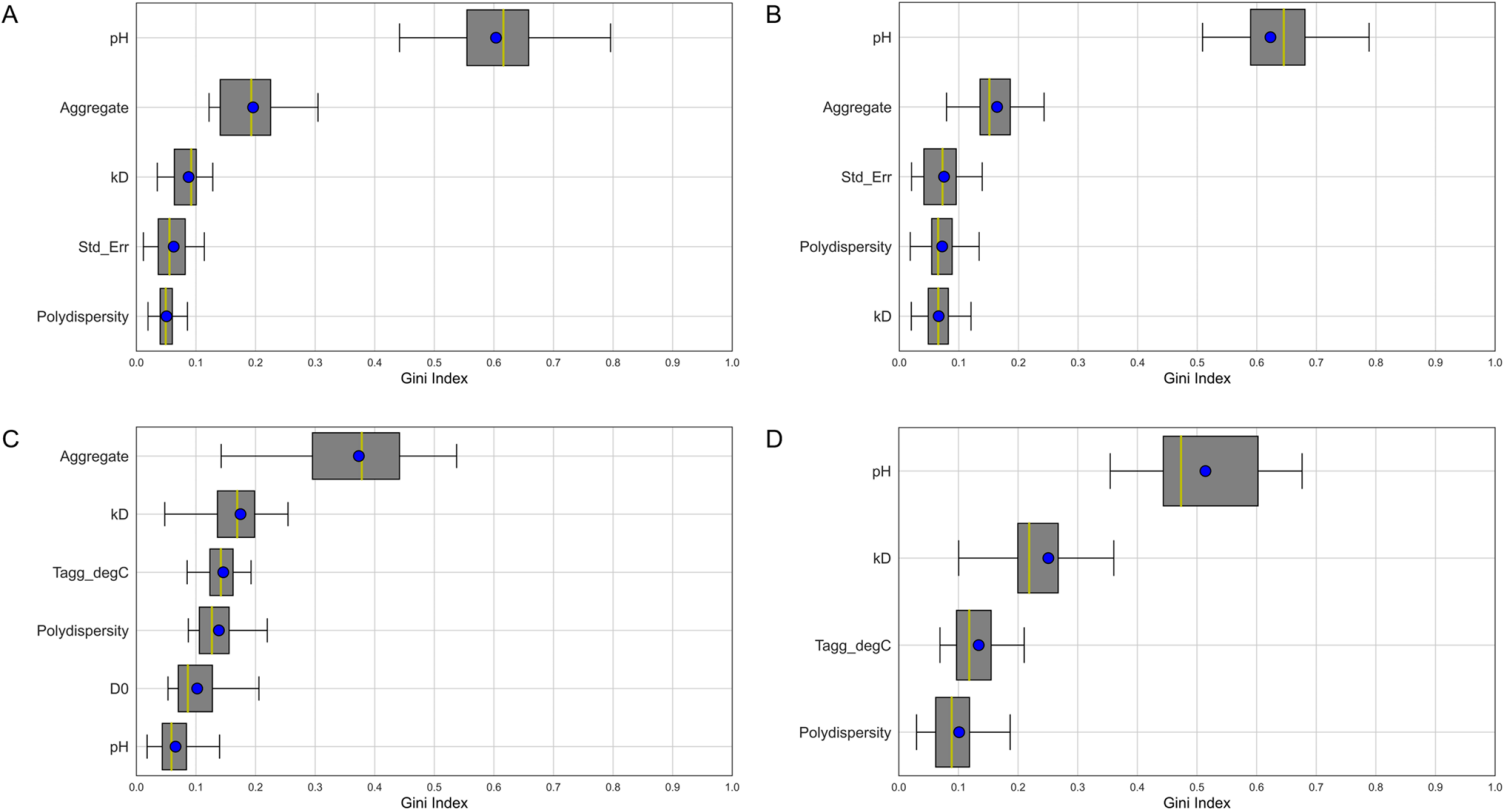
Importance of the features for each of the four ML models. (**A**) T_onset_ model; (**B**) T_m_1 model; (**C**) T_m_2 model; (**D**) T_m_3 model. For each model, the top features are ranked from most to least important (from top to bottom). Gini Index is a measure of the importance of the features; the higher the value the more important the feature is in the decision-making process of the model. For each feature, the box represents the interquartile range (Q1-Q3), the vertical green line inside it the median value, and the blue dot the mean value.

The selected models provide additional insights into the features that were most important for making the predictions (Fig. 7). When looking at the formulation parameters, the role of pH is very informative while the salt concentration is systematically ignored by all the models. In fact, by removing the salt concentration from the models’ inputs, as well as other metrics classed as less important such as Z-Average, improved accuracy in the predictions was obtained. On the other hand, metrics from both SEC and LS appear among the most important features in all the four models. Replicating the same results in the absence of inputs derived from either SEC or LS, results in a consistent deterioration of the accuracy. Importantly, this result demonstrates that the predictive information embodied in the DSC data is also contained in the data of other biophysical techniques that are likely already accessible or readily amenable to screening.

A better understanding of the relationships between the metrics, and therefore the assays used can also improve the understanding of the underlying biophysical mechanisms. The various input metrics are used differently by the four models in order to determine the target temperatures. For instance, the values of k_D_ are important predictors for T_m_3 but not for T_m_1. Likewise, the percentage of aggregation is an important predictor for the first three transitions but not for T_m_3. While more investigations are needed to better understand this difference, these early results might suggest that the biophysical mechanisms underlying the thermal transition points might be different.

The above results demonstrate that it is possible to predict certain metrics even with a dataset of limited size, with the potential to reduce the amount of sample and the time needed to perform measurements. It is worth mentioning that the mAbs used for this study were selected to be dissimilar based on previously published assay results [9]. While this choice provides a larger coverage of the developability space, it also presents a challenge for ML algorithms trying to learn the underlying relationships between metrics, which is what allows for better predictions. Nonetheless, cross-validation results showed that transition temperatures could successfully be predicted from limited data, and more measurement data can only improve this result. A local analysis based on the results of Fig. 6 confirms this hypothesis: Predictions are better in the region of higher density of data points (higher temperatures) whereas the largest errors are observed in regions where data is more sparse (lower temperatures).

The results across the four models reveal that predicting T_m_2 and T_m_3 seems to be more challenging than T_onset_ and T_m_1. Whereas this could be a consequence of the small dataset used for this study, in particular of the different shape that each temperature distribution has, it could also signal an intrinsic limitation of the features tested here: it is possible that metrics from LS and SEC can only partially explain the higher temperature transitions of mAbs observed with DSC, perhaps requiring additional metrics to complete the explanation. This is where XAI and expert knowledge can help by discerning the different contributions that the various metrics being tested bring towards the algorithmic decision-making process which generates the predictions. The expert user, on the other hand of the process, can better direct the experiments to identify missing metrics more quickly. While the former can be viewed as a retrospective feature selection mechanism (or *a posteriori*), the latter could be defined as a knowledge-based feature selection (or *a* *priori*), both aimed at improving what a model can learn and make it transparent. The study presented here offers then a tangible example of the benefits that can be obtained by opening the black box and allowing knowledge exchange with expert users in the process, which can ultimately lead to better designed experiments and optimisation of the resources.

## Conclusion

In case studies 1 and 2 we described new metrics that arise from combinations of analytical techniques and experimental conditions. With the new metrics we explain certain molecular liabilities such as domain-specific aggregation propensity and electrostatic attraction. We also showed how these molecular liabilities are linked to structural properties. In the future we envisage this knowledge being used in ML algorithms to embed the expert’s understanding of molecular behaviour into an explainable predictive model.

In case study 3 we further investigate computational approaches to predict common thermal stability metrics using biophysical parameters from other screening techniques as input parameters into machine learning models. This predictive approach can reduce the experimental effort of developability assessments and bring them earlier in development. Moreover, the ML methods we used give insights into key predictive parameters and allow knowledge exchange with expert users. These features can unlock better predictions that also come with a scientific explanation.

## Supplementary Information

Below is the link to the electronic supplementary material.

Supplementary file1 (PDF 219 kb)

Supplementary file2 (XLSX 27 kb)

Supplementary file3 (XLSX 23 kb)
