# Supplementary material for: Analytical Workflows to Unlock Predictive Power in Biotherapeutic Developability
Source: Pharm Res. 2022 Dec 5;40(2):487–500. doi: 10.1007/s11095-022-03448-y (PMC9944381; doi:10.1007/s11095-022-03448-y)
Supplement: Supplementary file 1 — Supplementary file1 (PDF 219 kb) [file 11095_2022_3448_MOESM1_ESM.pdf]

Supplementary Information

Analytical workflows to unlock predictive power in biotherapeutic developability

Markos Trikeriotis, Sergey Akbulatov, Umberto Esposito, Athanasios Anastasiou, Oksana I. Leszczyszyn

Table S1. Properties of the monoclonal antibodies used in this study

Name and abbreviation	Isotype	PDB ID[a]	Extinction coefficient[b]	Monomericity (%)[c]	Theoretical pI[d]		
					mAb	Fab	Fc
Atezolizumab (mAb-1)	IgG1	5X8L (Fab)	1.62	97.2	7.68	7.90	7.05
Golimumab (mAb-2)	IgG1	5YOY (Fv)	1.48	99.4	7.97	8.26	6.95
Ixekizumab (mAb-3)	IgG4	6NOV (Fab)	1.39	99.3	7.43	7.95	6.04
Lampalizumab (mAb-4)	IgG1	4D9Q (Fab)	1.41	98.8	6.67	6.22	7.11
Rituximab (mAb-5)	IgG1	2OSL (Fab)	1.64	98.4	8.12	8.48	7.11

[a] In parenthesis the fragment type that corresponds to each PDB structure. The Protein Data Bank can be found at: https://www.rcsb.org/

[b] Calculated by Evitria SA (L x g^{-1} x cm^{-1} at 280 nm)

[c] Reported by Evitria SA

[d] Calculated using Prot pi, Protein Tool (Zurich University of Applied Sciences; https://www.protpi.ch/Calculator/ProteinTool)

Primary sequences of the studied mAbs

Atezolizumab (mAb-1)

Heavy chain

EVQLVESGGGLVQPGGSLRLSCAASGFTFSDSWIHWVRQAPGKGLEWVAWISPYGGSTYYADSVKGRFTISADTSKNTAYLQ
MNSLRAEDTAVYYCARRHWPGGFDYWGQGTLVTVSAASTKGPSVFPLAPSSKSTSGGTAALGCLVKDYFPEPVTVSWNSGAL
TSGVHTFPAVLQSSGLYSLSSVVTVPSSSLGTQTYICNVNHKPSNTKVDKKVEPKSCDKTHTCPPCPAPELLGGPSVFLFPPKPKD
TLMISRTPEVTCVVVDVSHEDPEVKFNWYVDGVEVHNAKTKPREEQYASTYRVVSVLTVLHQDWLNGKEYKCKVSNKALPAPI
EKTISKAKGQPREPQVYTLPPSREEMTKNQVSLTCLVKGFYPSDIAVEWESNGQPENNYKTTPPVLDSDGSFFLYSKLTVDKSR
WQQGNVFSCSVMHEALHNHYTQKSLSLSPGK

Light chain

DIQMTQSPSSLSASVGDRVTITCRASQDVSTAVAWYQQKPGKAPKLLIYSASFLYSGVPSRFSGSGSGTDFTLTISSLQPEDFATY
YCQQYLYHPATFGQGTKVEIKRTVAAPSVFIFPPSDEQLKSGTASVVCLLNNFYPREAKVQWKVDNALQSGNSQESVTEQDSK
DSTYSLSSTLTLSKADYEKHKVYACEVTHQGLSSPVTKSFNRGEC

Golimumab (mAb-2)

Heavy chain

QVQLVESGGGVVQPGRSLRLSCAASGFIFSSYAMHWVRQAPGNGLEWVAFMSYDGSNKKYADSVKGRFTISRDNSKNTLYL
QMNSLRAEDTAVYYCARDRGIAAGGNYYYYGMDVWGQGTTVTVSSASTKGPSVFPLAPSSKSTSGGTAALGCLVKDYFPEPV
TVSWNSGALTSGVHTFPAVLQSSGLYSLSSVVTVPSSSLGTQTYICNVNHKPSNTKVDKKVEPKSCDKTHTCPPCPAPELLGGPS
VFLFPPKPKDTLMISRTPEVTCVVVDVSHEDPEVKFNWYVDGVEVHNAKTKPREEQYNSTYRVVSVLTVLHQDWLNGKEYKCK
VSNKALPAPIEKTISKAKGQPREPQVYTLPPSREEMTKNQVSLTCLVKGFYPSDIAVEWESNGQPENNYKTTPPVLDSDGSFFLY
SKLTVDKSRWQQGNVFSCSVMHEALHNHYTQKSLSLSPGK

Light chain

EIVLTQSPATLSLSPGERATLSCRASQSVYSYLAWYQQKPGQAPRLLIYDASNRATGIPARFSGSGSGTDFTLTISSLEPEDFAVYY
CQQRSNWPPFTFGPGTKVDIKRTVAAPSVFIFPPSDEQLKSGTASVVCLLNNFYPREAKVQWKVDNALQSGNSQESVTEQDSK
DSTYSLSSTLTLSKADYEKHKVYACEVTHQGLSSPVTKSFNRGEC

Ixekizumab (mAb-3)

Heavy chain

QVQLVQSGAEVKKPGSSVKVSCKASGYSFTDYHIHWVRQAPGQGLEWMGVINPMYGTTDYNQRFKGRVTITADESTSTAYM
ELSSLRSEDTAVYYCARYDYFTGTGVYWGQGTLVTVSSASTKGPSVFPLAPCSRSTSESTAALGCLVKDYFPEPVTVSWNSGALT
SGVHTFPAVLQSSGLYSLSSVVTVPSSSLGTKTYTCNVDHKPSNTKVDKRVESKYGPPCPPCPAPEFLGGPSVFLFPPKPKDTLMI
SRTPEVTCVVVDVSQEDPEVQFNWYVDGVEVHNAKTKPREEQFNSTYRVVSVLTVLHQDWLNGKEYKCKVSNKGLPSSIEKTI
SKAKGQPREPQVYTLPPSQEEMTKNQVSLTCLVKGFYPSDIAVEWESNGQPENNYKTTPPVLDSDGSFFLYSRLTVDKSRWQE
GNVFSCSVMHEALHNHYTQKSLSLSLG

Light chain

DIVMTQTPLSLSVTPGQPASISCRSSRSLVHSRGNTYLHWYLQKPGQSPQLLIYKVSNRFIGVPDRFSGSGSGTDFTLKISRVEAE
DVGVYYCSQSTHLPFTFGQGTKLEIKRTVAAPSVFIFPPSDEQLKSGTASVVCLLNNFYPREAKVQWKVDNALQSGNSQESVTE
QDSKDSTYSLSSTLTLSKADYEKHKVYACEVTHQGLSSPVTKSFNRGEC

Lampalizumab (mAb-4)

<u>Heavy chain</u>

EVQLVQSGPELKKPGASVKVSCKASGYTFTNYGMNWVRQAPGQGLEWMGWINTYTGETTYADDFKGRFVFSLDTSVSTAYL
QISSLKAEDTAVYYCEREGGVNNWGQGTLVTVSSASTKGPSVFPLAPSSKSTSGGTAALGCLVKDYFPEPVTVSWNSGALTSGV
HTFPAVLQSSGLYSLSSVVTVPSSSLGTQTYICNVNHKPSNTKVDKKVEPKSCDKTHTCPPCPAPELLGGPSVFLFPPKPKDTLMI
SRTPEVTCVVVDVSHEDPEVKFNWYVDGVEVHNAKTKPREEQYNSTYRVVSVLTVLHQDWLNGKEYKCKVSNKALPAPIEKTI
SKAKGQPREPQVYTLPPSREEMTKNQVSLTCLVKGFYPSDIAVEWESNGQPENNYKTTPPVLDSDGSFFLYSKLTVDKSRWQQ
GNVFSCSVMHEALHNHYTQKSLSLSPGK

<u>Light chain</u>

DIQVTQSPSSLSASVGDRVTITCITSTDIDDDMNWYQQKPGKVPKLLISGGNTLRPGVPSRFSGSGSGTDFTLTISSLQPEDVATY
YCLQSDSLPYTFGQGTKVEIKRTVAAPSVFIFPPSDEQLKSGTASVVCLLNNFYPREAKVQWKVDNALQSGNSQESVTEQDSKD
STYSLSSTLTLSKADYEKHKVYACEVTHQGLSSPVTKSFNRGEC

Rituximab (mAb-5)

<u>Heavy chain</u>

QVQLQQPGAELVKPGASVKMSCKASGYTFTSYNMHWVKQTPGRGLEWIGAIYPGNGDTSYNQKFKGKATLTADKSSSTAYM
QLSSLTSEDSAVYYCARSTYYGGDWYFNVWGAGTTVTVSAASTKGPSVFPLAPSSKSTSGGTAALGCLVKDYFPEPVTVSWNS
GALTSGVHTFPAVLQSSGLYSLSSVVTVPSSSLGTQTYICNVNHKPSNTKVDKKVEPKSCDKTHTCPPCPAPELLGGPSVFLFPPK
PKDTLMISRTPEVTCVVVDVSHEDPEVKFNWYVDGVEVHNAKTKPREEQYNSTYRVVSVLTVLHQDWLNGKEYKCKVSNKAL
PAPIEKTISKAKGQPREPQVYTLPPSREEMTKNQVSLTCLVKGFYPSDIAVEWESNGQPENNYKTTPPVLDSDGSFFLYSKLTVD
KSRWQQGNVFSCSVMHEALHNHYTQKSLSLSPGK

<u>Light chain</u>

QIVLSQSPAILSASPGEKVTMTCRASSSVSYIHWFQQKPGSSPKPWIYATSNLASGVPVRFSGSGSGTSYSLTISRVEAEDAATYY
CQQWTSNPPTFGGGTKLEIKRTVAAPSVFIFPPSDEQLKSGTASVVCLLNNFYPREAKVQWKVDNALQSGNSQESVTEQDSKD
STYSLSSTLTLSKADYEKHKVYACEVTHQGLSSPVTKSFNRGEC

Salt Effect calculations

To generate the salt effect plot the change on the $T_{agg}2$ and T_m values must be calculated to give $\Delta T_{agg}2$ and ΔT_m respectively. The aggregation temperature shift, $\Delta T_{agg}2$, was calculated as:

$\Delta T_{agg}2 = T_{agg}2$ (low salt) - $T_{agg}2$ (high salt)

where $T_{agg}2$ (low salt) and $T_{agg}2$ (high salt) refer to the value of $T_{agg}2$ at NaCl concentration 0 mM and 150 mM respectively.

The unfolding temperature shift, ΔT_m includes the combined shift of the $T_m(C_H2)$, $T_m(C_H3)$ and $T_m(Fab)$, and was calculated as:

$\Delta T_m = |\Delta T_m(C_H2)| + |\Delta T_m(C_H3)| + |\Delta T_m(Fab)|$

where the ΔT_m for each domain or region was the difference between the respective T_m at NaCl concentration 0 mM (low salt) and 150 mM (high salt); for example for C_H2:

$\Delta T_m(C_H2) = T_m(C_H2)$(low salt) - $T_m(C_H2)$(high salt)

All the results of these calculations are listed on Table S2 below.

Table S2. Salt effect results for all mAbs

mAb	pH	ΔT_m (°C)	$\Delta T_{agg}2$ (°C)
mAb-1	4	13.2	11.0
mAb-1	5	8.1	8.3
mAb-1	6	3.5	1.5
mAb-1	7	2.2	-0.9
mAb-1	8	1.9	0.3
mAb-2	4	12.2	13.5
mAb-2	5	4.2	12.8
mAb-2	6	2.0	0.7
mAb-2	7	0.5	-2.6
mAb-2	8	1.1	-2.8
mAb-3	4	12.1	15.8
mAb-3	5	3.9	8.7
mAb-3	6	2.3	-2.9
mAb-3	7	1.9	-7.0
mAb-3	8	2.3	-5.7
mAb-4	4	9.8	17.9
mAb-4	5	4.9	1.9
mAb-4	6	4.0	N/A[a]
mAb-4	7	2.7	N/A[a]
mAb-4	8	7.0	-1.5
mAb-5	4	9.8	15.3
mAb-5	5	5.6	12.5
mAb-5	6	2.5	1.9
mAb-5	7	0.6	-1.2
mAb-5	8	0.4	-1.6

[a] The $\Delta T_{agg}2$ for this sample was not possible to determine because it was heavily aggregated at low salt conditions

Additional Supplementary Files

ESM_2.xls Input parameters for Machine Learning

ESM_3.xls Biophysical characterisation results for the 5 mAbs
